# Prolonged hypothermic storage of oocytes of the European common frog *Rana temporaria* in a gas mixture of oxygen and carbon monoxide

**DOI:** 10.1371/journal.pone.0288370

**Published:** 2023-07-20

**Authors:** Evgeniy Gagarinskiy, Viktor Uteshev, Eugeny Fesenko

**Affiliations:** Institute of Cell Biophysics RAS – A Separate Subdivision of Federal Research Centre “Pushchino Scientific Centre for Biological Research RAS”, Moscow, Russia; Friedrich-Loeffler-Institute, GERMANY

## Abstract

The maximum hypothermic storage time of amphibian oocytes is several hours, which is due to the peculiarities of the structure of the cell envelope. The authors of this paper have already demonstrated the possibility of increasing the storage period of unfertilized oocytes of the common frog (*Rana temporaria*) up to 5–7 days. The aim of the current study was to determine the possibility of using a 6.5 atm gaseous mixture of carbon monoxide and oxygen, for prolonged hypothermic preservation of unfertilized oocytes for 4 to 12 days. After four days, oocytes stored under CO+O_2_ conditions exhibited fertilization and hatching rates that were 1.6 and 2.2-fold higher than control, respectively. While no oocytes in the control group survived to day twelve, oocytes held under CO +O_2_ gas exhibited a 39±14% (38 out of 99 oocytes in total) fertilization rate, however only 1±2% (1/99) of those hatched. This approach is promising for the storage of genetic material from female amphibians, particularly in respect to managing and restoring endangered species, but may also be applicable to oocytes of other classes of vertebrates.

## 1. Introduction

In recent years, the rate of extinction of the class Amphibia has increased by more than 200 times over the historic background rate and is attributed to numerous abiotic and biotic factors [1]. Almost one-third of amphibian species are on the edge of extinction, which is the highest value among all the vertebrates [2, 3]. Reproductive technologies developed for amphibia make it possible to slow down the alarming rate of species loss and genetic loss within species, by facilitating breeding events designed to enhance reproductive output and genetic diversity. Assisted reproductive technologies include hormone-induced methods of obtaining gametes (sperm and ovulating oocytes) and artificial conception. However, synchronization of spermiation and ovulation from live animals is challenging, and frequently long term storage of gametes from one sex or the other becomes necessary. However, the techniques for long-term ultra-low temperature (-196°C) storage of ovulated amphibian oocytes have yet to be developed, primarily due to the higher complexity of oocytes compared to sperm cells and the presence of barriers with low permeability that isolate them from the environment where they are developed [4]. Additionally, the macroscopic size of amphibian’s oocytes makes them generally slow to equilibrate with standard cryoprotectants for effective vitrification, which is also the case for embryos.

In the absence of cryopreservation and ultra-low storage methodologies for amphibian oocyte, short-term cold (+4°C) storage of unfertilized eggs remains of critical interest. Recently, the longevity of oocyctes has been extended from only a few hours [5–8] to 5–7 days by isolating the oocytes in small sealed containers [9, 10]. While the sealed caps prevented the oocytes from drying, the lack of direct contact with aqueous solutions prevented chemical compositional changes in the egg jelly. The possibility of further prolongation of the storage period of oocytes at low positive temperatures is of great interest.

One approach with potential to increase the storage time of gametes in poikilothermic animals is the use of biologically active gases like carbon monoxide (CO) and oxygen (O_2_). Carbon monoxide is particularly interesting, as it can interact with cell receptors and protects the cell through vasoactive, anti-proliferative, antioxidative, anti-inflammatory, and anti-apoptotic action [11–13]. Carbon monoxide easily penetrates cell membranes and quickly diffuses through tissues. In a large number of studies, it was shown that oxygen persufflation has the ability to increase storage time of donor organs [14–16]. Hyperoxia facilitates the diffusion of oxygen into the cell, activates oxidative phosphorylation, increases the synthesis of high-energy compound and reduces lactose levels in cells [17]. The combined use of CO and O_2_ has proven to be highly effective in prolonging the storage time of warm-blooded animal organs by four to six times when compared to traditional hypothermal preservation techniques using specialized solutions [18–21].

The goal of the current study was to investigate the possibility of applying a preserving gas mixture based on carbon monoxide and oxygen for hypothermal preservation of unfertilized oocytes of the common frog and to increase the hypothermal storage period to 12 days.

## 2. Material and methods

### 2.1 Animals

Adult male and female common frogs *R*. *temporaria* were collected during the wintering period in December in the Moscow Region. The weight of the animals was 42 ± 2.5 grams for males and 52 ± 3 grams for females. The frogs were kept in plastic boxes (60×40×20 cm) with a small amount of water (depth of water was 5 cm, 10 animals per box). Male and female frogs were kept separately. The boxes were stored in a dark cold room at 4°C; water was refreshed every week. The study was conducted in accordance with the requirements of Directive 2010/63/EU on the protection of animals used for scientific purposes. The experimental protocol was validated by the Ethical Committee of ICB RAS (Approval ID: 1/092021, date: 2021-9-08).

### 2.2 Obtaining oviduct oocytes

In order to obtain oviduct oocytes, each female frog was taken from the cool box and injected with the gonadotropic hormone “Surfagon” (Synthetic analogue of Gonadotropin-releasing hormone (GnRH)) (Mosagrogen, Russia) The hormone was administered intraperitoneally, in a volume of 0.1 ml 50 μg per animal. After injection, each female frog was placed into an individual box (15×15×15 cm) with a depth of 5 cm water. Each frog was held at 17°C for 36–48 hours, and then a sample of ovulating oocytes (min. of 70 cells) was collected by gentle massage of the abdomen. The first 10–15 oocytes were not used in the experiment.

### 2.3 Obtaining urinal sperm

Each male frog was taken from the cool box and injected with “Surfagon” (0.1 ml / 25 μg per animal). At least three males were used for each experiment. After the injection, the male frog was placed into an individual box (15×15×15 cm, 5 cm water) and held at 17°C. At 1, 3, and 6 hours after injection, urinal sperm was sampled by gentle massage of the abdomen. The urinal sperm was collected into an Eppendorf tube and placed in a refrigerator. Immediately after receiving each sperm sample, we determined the concentration and motility of spermatozoids with a Goryaev counting-chamber device. Portions with at least 60–120·10^6^ cells·ml^-1^ concentration and 75% motility were used for the mix preparation: 1, 3, and 6—hour samples from one male were mixed together. The sperm samples from an individual male were split into two 0.25 mL aliquots, one for the experimental group and the other for the control. The sperm samples were used for oocyte fertilization immediately after the mix preparation. The male frog was brought back to the cooling box (+4°C).

### 2.4 Oocytes storage

**Fig 1:**
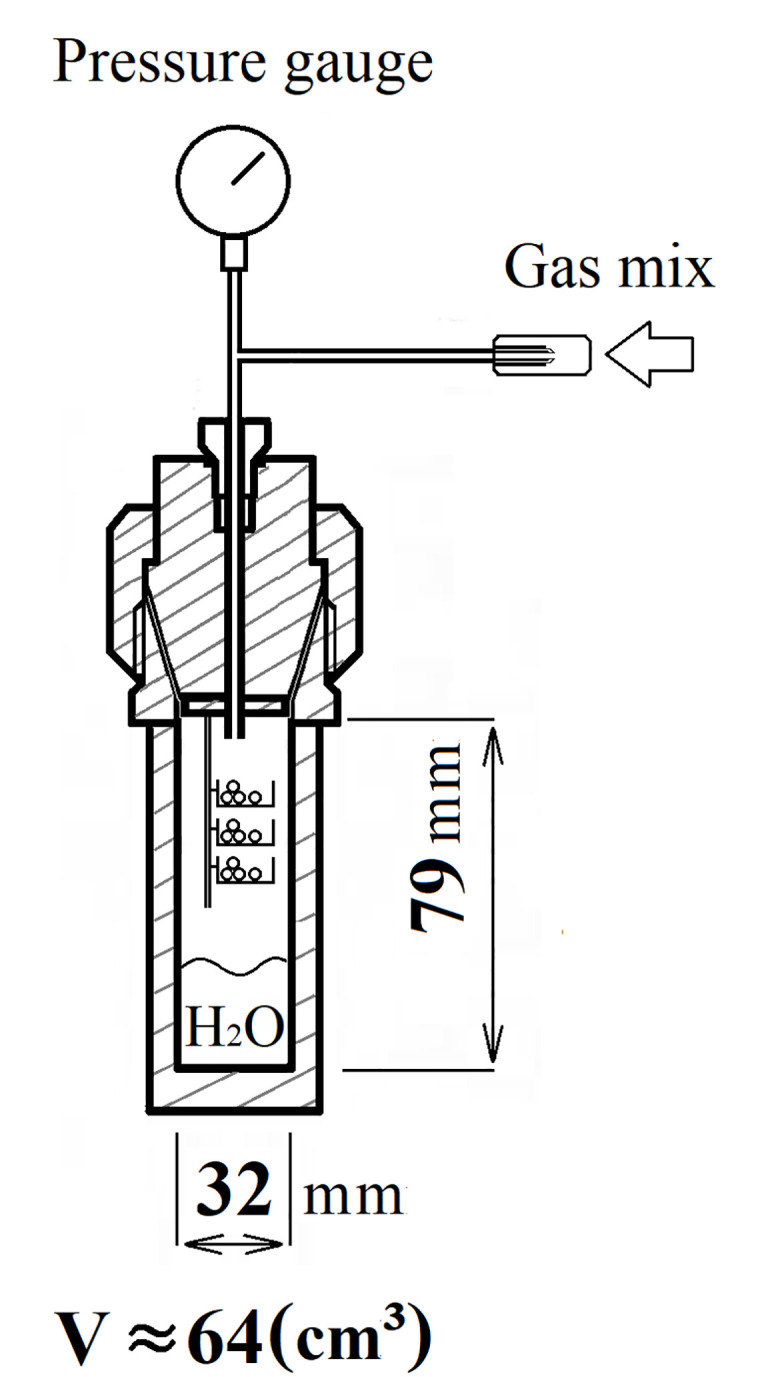
The scheme of the chamber for hypothermal storage of frog oocytes in the atmosphere of biologically active gases.

The oocytes were stored in a special chamber for gas preservation based on a steel chemical reactor Vivor (Premex, Switzerland). Prior to the experiment, the chamber was cooled to +4°C. To maintain proper humidity, distilled water was poured to a level of 1 cm. The oocytes (min. 10 per group) were placed on pieces of foiled substrate 15 mm in diameter. Three pieces of substrate, each with at least 10 oocytes, were individually placed on custom made platforms inside the chamber (Fig 1). To replace the air, the chamber was purged with a preserving gas mixture composed of oxygen and carbon monoxide in a 1:1 ratio. The experimental gas mixture was prepared by mixing the separate components in a 5 L oxygen balloon. The purity of the used gases was at least 4.0 (99.99%). Then the lid of the chamber was tightly sealed, and the gas mixture was pumped into the chamber up to the excessive pressure value of 6.5 atm. To stabilize the temperature during storage, the chambers with oocytes were immersed in a container filled with water cooled down to +4° C. The container was then placed in the refrigerator at +4°C.

The stored oocytes were divided into four experimental groups. Group 1 was stored at a temperature of +4°C for 1 day, group 2 for 4 days, group 3 for 7 days, and group 4 for 12 days. After the incubation of each set of oocytes for the designated time period, the chambers were taken from the refrigerator, and the pressure was gently released over 10 minutes until reaching atmospheric pressure. All the work with the gas mixture was carried out in a ventilated laboratory cabinet equipped with carbon monoxide sensors. Control oocytes, collected at the same time, were stored in a Vivor chamber under normal atmospheric pressure (air) for 1, 4, 7, or 12 days.

### 2.5 Fertilization

The substrates with oocytes taken from the chamber were placed into Petri dishes (90 mm diameter, 10 oocytes per dish), and 1 sample of urinal sperm (0.25 ml) was added to each plate. The sperm was removed after 15 minutes of incubation (oocytes were rinsed with three portions of 5 ml water), the oocytes were covered with settled water (tap water that has been left in an open container for 24 hours before being used in the experiment), and the fertilized eggs were kept for development until the stage of hatching of larvae. The fertilization rate was assessed by the emergence of the first cleavage of the oocytes, ~3–3.5 hours. The assessment of fertilization and subsequent development of the embryos was carried out using a Leica MZ16A stereomicroscope (Leica Microsystems, Germany). The percentage of fertilized oocytes was calculated by dividing the number of oocytes at the first cleavage stage by the total number of oocytes used in the experiment. The percentage of embryos that hatched (free swimming after release from the embryonic membrane) was calculated by the ratio of the larvae number to the initial number of oocytes. In native control experiments, the quality of oocytes was estimated for each female frog in the study by testing the fertilizing capacity of a subset of the oocytes immediately after collection (0 days of storage). The stages of embryonic development of *R*. *temporaria* were assigned according to Dabagyan V.N. (1975) [22].

Data analysis was performed using SigmaPlot vs. 12.5 (Systat Software Inc., USA); the data were represented as the mean value ± standard deviation. The significance of the differences was determined by the Mann-Whitney U-test.

## 3. Results

**Fig 2:**
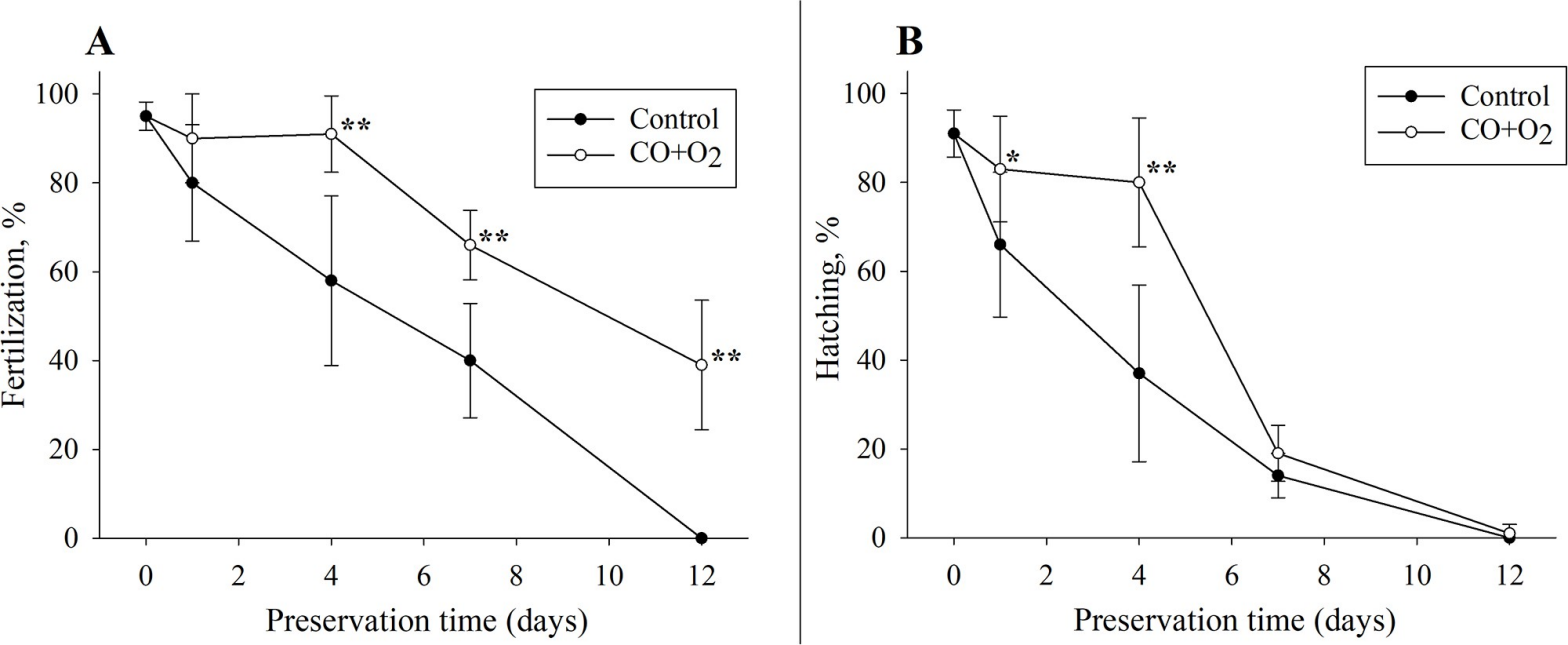
Percentage of fertilized eggs from the overall oocyte number (A) and percentage of hatched larvae from the overall number of eggs (B) after storage at +4°C. Each graph point represents a mean value ± SD for frogs (min. 30×3 oocytes). Day 0 –fresh oocytes. The asterisks represent the reliability of differences between the experimental groups and the control group at the same time point of hypothermal preservation: * P = 0.05, ** P = 0.01.

Fertility of control oocytes after storage in the chamber without gas mixture at 4°C declined steadily overtime, reaching 58±19% (58/98 oocytes) and 41±12% (37/91 oocytes), after four and seven days, respectively. By day 12 of storage, no eggs capable of being fertilized were found in control. However, the fertility of oocytes stored at 4°C in carbon monoxide: oxygen gas mixture after day 4 in storage was similar (91+ 9%) (101/111) to that of the freshly collected eggs on day 0 (95+4%) (150/158). Fertilizability decreased to 66+8% (60/91) by day 7 but was still 39+14% (38/99) on day 12. (Fig 2A).

The percent hatching in the control group decreased from 91±5% (144/158) to 37±19% (38/98) by day 4 of storage, and to 13±5% (12/91) by day 7 of storage. In the experimental group, the hatching ratio was similar (P<0.01) between the fresh oocytes and those fertilized on day 4 (80±15%) (88/111). However, a dramatic decrease in hatching rate to 19±6% (17/91) was observed in the experimental oocytes that were fertilized on day 7 compared to day 4. After 12 days of incubation, hatching percentage comprised only 1±2% (1/99) (Fig 2B).

## 4. Discussion

Long term storage of amphibian oocytes is challenging for numerous reasons. Not only are there no successful attempts to date for low-temperature cryostorage [23], but short term cold storage is complicated by rapid chemical changes to the egg-jelly envelope in response to water, forming the barrier to polyspermy within 10–30 minutes of ovaposition [6, 24, 25]. It has been demonstrated that use of hypertonic saline (SAR) slows down the processes of egg-jelly degradation, prolonging the storage of oocytes at 4°C for up to 4–8 hours. [6, 7, 26].

**Fig 3:**
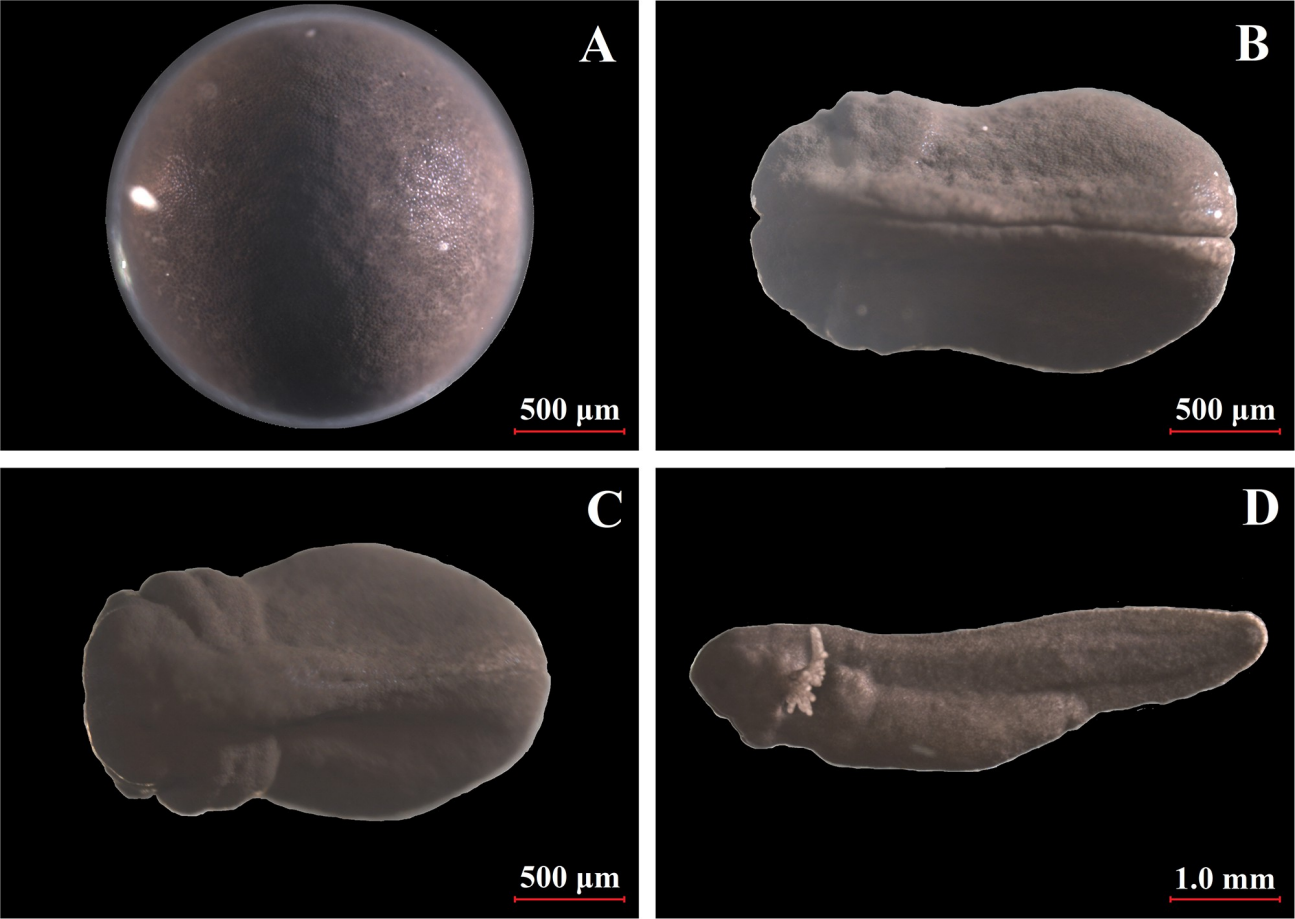
Stages of development of *R*. *temporaria* zygote after storage at 4°С in the atmosphere of biologically active gases CO+O_2_ for 4 days. Stages of embryonic development: A. Stage 10 (21 h); B. Stage 22 (47.5 h); С. Stage 24 (53.5 h). Larval development: D. Stage 31 (4.5 days).

The current study aimed to combine the “sealed containers” approach based on the preservation of oocytes without the use of storage solutions [9, 10] and the application of protective properties of biologically active gases, particularly, a mixture of carbon monoxide and oxygen. We observed a steady decrease in fertility and hatching parameters for oocytes stored in sealed containers from 1–12 days under air or CO + O_2_ gas; however, the number of hatched larvae was significantly higher for the oocytes stored under the gas mixture compared to control (Fig 3). The analysis of oocytes after fertilization did not show any specific tendencies of arrested embryonic development in the experimental group compared to the control.

It is well known that for amphibians, ovulated oocytes that remain unfertilized undergo rapid degradation, which is referred to as post-ovulatory oocyte aging [27, 28]. During incubation at room temperature, many apoptotic events are spontaneously triggered, including release of mitochondrial cytochrome c, caspase activation and fragmentation of the nucleus [29, 30]. Pasquierа and Tokmakov showed that the unfertilized oocytes of *X*. *laevis* die in 48–72 hours after laying due to apoptosis. Specific apoptotic features were observed in the unfertilized eggs, namely release of cytochrome c, morphological changes of nuclei, increase in the ADP to ATP ratio and exhaustion of the ATP pool [31]. Similar apoptotic processes have been detected in the eggs of other vertebrates (fish, mammals) and some invertebrates (sea urchins, starfish) [31]. In the studies by Johnson (2010), microinjection of cytochrome c into *Xenopus* oocytes reliably induced apoptosis which was reflected in a change in oocyte morphology after several hours and the appearance of indirect markers of caspase activation [32].

Carbon monoxide affects cell function by binding the structures containing transient metal ions (for example, Fe, Cu), the most well-known of which is heme. A large number of proteins contain heme (for example, soluble guanylyl cyclase (sGC), cyclooxygenase, cytochrome P450, cytochrome c-oxidase, inducible NO-synthase (iNOS)). The presence of СО donors (Carbon monoxide-releasing molecules CORM-2 (tricarbonyldichlororuthenium(II) dimer), CORM-A1 (sodium boranocarbonate) significantly decreases the number of porcine oocytes undergoing apoptosis *in vitro* [26]. Direct anti-apoptotic action of CO on mitochondria realized via decrease of mitochondrial membrane permittivity was demonstrated for the first time in a study of primary astrocyte cultures [33]. Cytochrome c initiates apoptosis when released from the mitochondria into the cytosol where it binds to apoptotic protease activation factor (APAF1), which triggers a caspase cascade and subsequent apoptotic reactions. Additionally, cytochrome c is able to catalyze lipid peroxidation reactions, acquiring peroxidase-like properties after a conformational change upon binding to cardiolipin which is a phospholipid of the inner mitochondrial membrane [34]. Kapetanaki et al. demonstrated that CO binds to cytochrome c and inhibits the decrease in its reduction potential and subsequent peroxidase activity. Thus, CO inhibits the oxidation of cardiolipin that normally leads to increased permeability of the outer mitochondrial membrane initiating the apoptotic cascade. [35].

Finally, CO also increases reactive oxygen species (ROS) production by binding to mitochondrial cytochrome c-oxidase and/or NAD(P)H-oxidase of the plasma membrane. CO-induced ROS enhances mitochondrial biogenesis by activating transcription factors, such as nuclear respiratory factor-1 (Nrf-1), Nrf-2 and the gamma-coactivator-1α [36]. The estimated number of mitochondria under normal conditions is at least 115 million per mature oocyte of *R*. *temporaria* (Romek and Krzysztofowicz, 2005). Mitochondrial biogenesis allows the cell to replace damaged mitochondria and manage the periods of elevated metabolic requirements [34]. One can suppose that CO-mediated enhancement of mitochondria biogenesis prevents the internal mechanisms of amphibian oocyte degradation, or arrests embryo development, in unfavorable conditions when the deficiency of nutrients is accompanied by a decrease in the number of normal functionally and actively respiring mitochondria [36, 37]. Thus, the positive effects of СО on prolongation of hypothermal storage of amphibian oocytes could be related to protection from apoptotic mechanisms as well as enhanced mitochondrial biogenesis.

The concentration of carbon monoxide varies significantly between individual studies, ranging from a few parts per million to elevated levels achieved by a pressure of 2–4 atm. In most studies assessing the protective functions of carbon monoxide, gas concentrations within 250–1000 ppm are used to avoid potential negative effects on the body associated with toxic exposure [38]. However, in studies on long-term storage of isolated organs, empirically determined high concentrations of CO up to several atmospheres are used [19]. We took the latter approach in our experiments.

The second component of the gas mixture, oxygen, is known to have a positive effect on the preservation of organs during temporary storage [39]. In previous research by Gurin et. al., [40] it was assumed that the protective effect of oxygen was due to the maintenance of a reduced, but stable level of aerobic metabolism during hypothermic storage. Maintenance of the latter does not allow cells to switch to anaerobic respiration, accompanied by accumulation of lactic acid, a decrease in ATP production, which would normally result in a failure of ATP dependent Na/K pumps and Ca pumps follows by osmotic swelling of the cell and eventual lysis. We found no data on biological effects of oxygen on the preservation of amphibian eggs, however, there are reports of prolonged survivability of amphibian sperm under conditions of aeration with oxygen [9, 41, 42].

In this study, we did not assess the efficacy of using individual gases O_2_ and CO for oocyte preservation. Instead, our methodology was guided by earlier investigations conducted on rat hearts, which demonstrated that pure oxygen preservation led to inferior organ function compared to a gas mixture of carbon monoxide and oxygen (CO + O_2_). Furthermore, utilizing pure carbon monoxide for preservation resulted in organ death (data unpublished).

The prolongation of the amphibian oocyte storage time could significantly improve the logistics and facilitate the exchange of genetic material between scientific institutions, by eliminating the requirement for the transportation of animal specimens for mate pairing. Transporting animals is usually accompanied by an increase in stress on the organism, and in some cases leads to death, and is particularly undesirable when managing populations of endangered species. The capacity to prolong oocyte viability for hours or days provides a much broader window for obtaining viable sperm samples from males, and directing specific genetic pairings in IVF attemtps.

The approach of using a mixture of CO and O_2_ gas for the preservation of amphibian oocytes could potentially be applied to oocytes of other classes of vertebrates as well. For example, sturgeon fish have similar embryonic development to that of *R*. *temporaria* and our proposed method of hypothermic storage under a mixed gas atmosphere could be transferable to sturgeon eggs, which makes the attempt to adapt the proposed method to prolonged hypothermic storage of sturgeon eggs very relevant.

## Supporting information

S1 File (XLSX)
